# Global hotspots and trends in research on preschool children’s motor development from 2012 to 2022: a bibliometric analysis

**DOI:** 10.3389/fpubh.2023.1118674

**Published:** 2023-06-02

**Authors:** Jun-Wei Wang, Sha Qu, Zhi-Cheng Zhu, Xing Zhao, Wen-Jing Song, Xue Li, Wan-Di Chen, Dong-Mei Luo

**Affiliations:** ^1^School of Sports Science, Beijing Sport University, Beijing, China; ^2^School of Sport Medicine and Health, Chengdu Sport University, Chengdu, China; ^3^Academic Administration, Chengdu Sport University, Chengdu, China

**Keywords:** preschool children, motor development, bibliometrics analysis, CiteSpace, research hotspots, research trends

## Abstract

**Background:**

Motor development plays an important role in human development throughout the lifespans, from conception to death, and has received increasing scholarly attention in recent years. However, valuable comprehensive reviews and literature analysis on this topic are still lacking. Here, this bibliometric study aimed to identify global motor development research hotspots and trends on preschool children’s motor development from 2012 to 2022.

**Methods:**

CiteSpace 6.1.R4 was used to visualize and analyze general bibliometric characteristics, research hotspots, and trends through a review of 2,583 articles on the motor development of preschool children, which were published from 2012 to 2022 and included in the Web of Science Core Collection.

**Results:**

Research on motor development in preschool children has been carried out into a phase of rapid development. The top five frequently occurring keywords were physical activity (n = 489), performance (*n* = 319), intervention (*n* = 222), health (*n* = 196), and executive function (*n* = 165); The top five keywords in terms of centrality are academic achievement (0.22), low birth weight (0.16), association (0.14), brain (0.13), and cerebral palsy (0.13). Thirteen keyword clusters were produced from the log-likelihood ratio (*Q* = 0.74, *S* = 0.88), and five research topics has been received focused attention in recent years. The keywords with the strongest citation bursts in the last 5 years are developing country (*S* = 5.92), school-aged children (*S* = 5.86), middle-income country (*S* = 3.46), efficacy (*S* = 5.41), readiness (*S* = 3.21), motor proficiency (*S* = 3.6), and screen time (*S* = 3.3), indicating newly emerging research trends.

**Conclusion:**

The results indicated that interventions involving fundamental movement skills, cognitive function, 24-h movement behaviors, neurodevelopmental disorders, and health-related fitness were hot topics in the field of motor development over the last decade. Emerging research trends generally center on school readiness, socioeconomic status, motor proficiency, and screen time.

## 1. Introduction

Motor development refers to changes in movement patterns that occur over an individual’s lifespan, as well as the process through which these changes take place (1). Gabbard’s “developmental continuum of life-span motor behavior” outlines three distinct phases of motor development during childhood and adolescence (2). The first phase is the fundamental movement phase, which is characterized by the acquisition of fundamental motor skills (FMS). FMS serve as the foundation for more complex movement patterns that are required in sports, games, and other physical activities, such as object control, locomotor, and stability skills (3), so this phase is considered a significant milestone in movement development during early childhood (ages 2–6). The second stage is the sport skill phase, which occurs from mid-childhood to late childhood and involves progressively improving children’s motor skills (MSs) and motor awareness abilities to adapt sports and recreational activities in a variety of settings (3). The third and final phase is the growth and refinement phase, which occurs from late childhood to adulthood and allows for the consolidation of learned motor skills (4).

Motor skills, which include gross and fine motor skills and motor coordination, comprise a series of fluid and effective movements learned for the acquisition of specific skills (3). These skills are deployed in almost every aspect of daily life and are a crucial component of children’s overall development; FMS provides an important foundation for the development of motor skills. Thus, motor development is a crucial aspect of human development throughout the life span, and has received increasing scholarly attention in recent years due to its profound impacts on physical fitness, academic performance, and psychological functioning (5, 6).

Health characteristics during the preschool years, which is a crucial period for individual growth and development, has a significant bearing on subsequent development throughout the lifespan (7). This phase constitutes the peak and the most sensitive period of motor development, with studies have shown a moderate to strong positive correlation between the level of motor development and the physical fitness, cognitive level, lifelong sports aptitude, and social and personality development of preschoolers (8–10).

In recent years, there has been a surge in research on motor development, with more than 200 publications being produced annually and new research paradigms being continuously cited in this field. Consequently, research hotspots and trends in this field have evolved, posing a formidable analytical challenge for researchers. However, a comprehensive review of the literature in this field is still lacking, and new research methods are urgently required for the analysis of research hotspots and trends.

Bibliometrics is an interdisciplinary field that applies mathematical and statistical methods to perform quantitative analyses of various knowledge repositories (11). As a result, researchers can extract the information they require from the vast amount of available literature, identify research hotspots and development trends in a particular field, and thus determine the future developmental direction of research (12). Bibliometric methods have been widely applied in fields such as medicine and kinesiology using multiple databases (13, 14). However, their use in the interdisciplinary field of movement development research has been more limited, and the research hotspots and trends in this field have not been fully described.

Therefore, this review focuses on the studies published during the period from 2012 to 2022 about preschool children’s motor development and included in the Web of Science Core Collection (WoSCC). Bibliometric and visual analyses are used to explore the general metrological characteristics, research hotspots, and development trends in this field, as quantitative analysis is currently lacking. The aim is to provide inputs and recommendations for future research in this field.

## 2. Materials and methods

### 2.1. Data sources and search strategies

We conducted a search for the articles published from 2012 to 2022 in the WoSCC database using the search terms listed in Table 1. The article type was set to “article” and “review,” and “English” was selected as the language of the articles. The search and articles download were completed in October 2022. In total, 2,583 articles were included in the review.

**Table 1:** Search strategies.

Step	Search format	Results
#1	TS = (preschooler* OR “preschool children” OR “early childhood” OR kindergarten OR “Preschool Students” OR “Nursery School Students” OR “young child” OR “2 ~ 5 years” OR “3 ~ 6 years”)	75,236
#2	TS = (“motor skill*” OR “gross motor” OR “fine motor” OR “motor development” OR “developmental motor” OR “fundamental motor skill*” OR “FMS” OR “locomotor skill*” OR “Object skill*” OR “balance skill*” OR “one-foot balance” OR coordination OR “motor coordination” OR “hand-eye coordination” OR “developmental coordination” OR “visual-motor coordination” OR “posture control” OR “control of posture” OR “motor competence” OR “motor proficiency” OR “motor ability*” OR “motor fitness” OR “motor difficulty*” OR “motor delay*” OR “perceived competence” OR “motor impairment*” OR “movement assessment”)	264,915
#3	#1 AND #2	2,583

### 2.2. Data downloading and pre-processing

The data extraction process was carried out by two researchers, who searched for, downloaded, and validated the articles. The authors also discussed any issues and collectively standardized the results. The extracted information included the names of the authors, article titles, full abstracts, publication sources, and cited references, which were downloaded in plain text format, and imported into the CiteSpace 6.1.R4 software. Data pre-processing primarily consisted of filtering and removal of duplicate data.

### 2.3. Bibliometrics and visualization analysis

We used CiteSpace 6.1.R4 to perform scientific collaboration network analysis, co-citation analysis, co-occurrence network analysis, and citation and keyword burst analysis on the content of the included literature. The analyses were aimed at identifying general bibliometrics, research hotspots, and developmental trends in the field (15). We visualized the data using GraphPad Prism 7.0 to plot the number of publications and citation frequency from 2012 to 2022. Binomial regression was used to predict the growth trend, and we determined journal impact factors using the Journal Citation Reports published by Clarivate Analysis in June 2022.

## 3. General bibliometrics analysis

### 3.1. Annual publication of articles and growth trends

**Figure 1:**
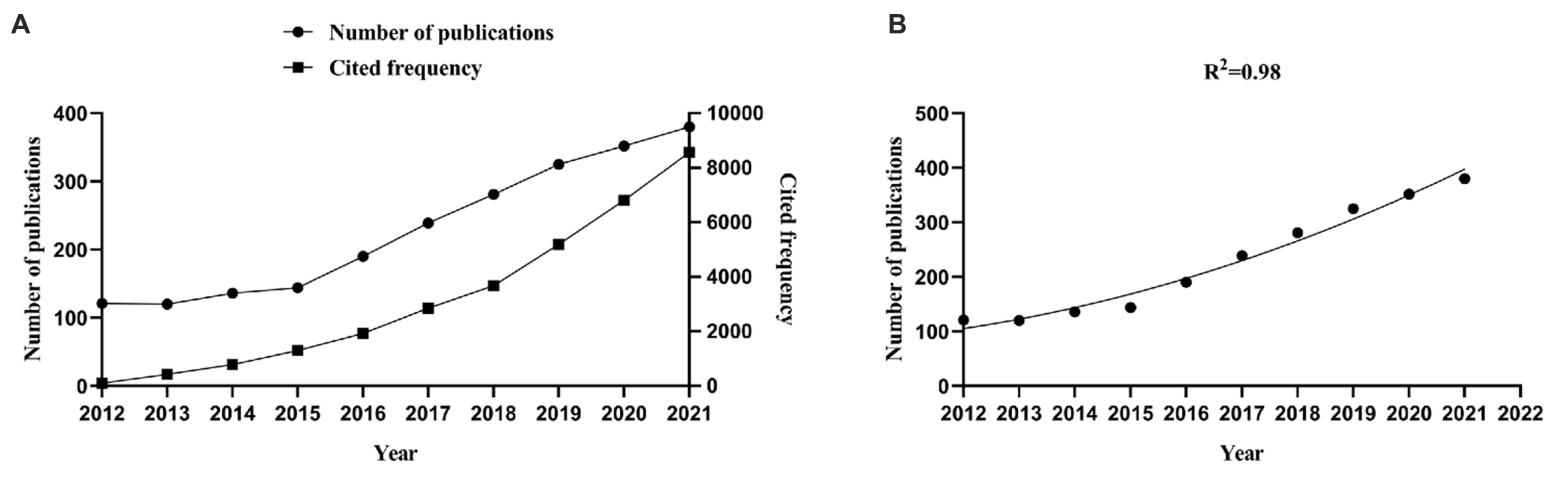
Number of publications and frequency of citations. The number of annual publications and citations **(A)**; the binomial fit curve of growth trends **(B)**.

We conducted a bibliometric analysis of 2,583 studies included in the review. The international research on preschool children’s motor development can be divided into two phases according to the characteristics of articles published from 2012 to 2021. The first phase, spanning from 2012 to 2015, was a period of slow-paced growth, with more than 120 annual publications and a gradual increase in citation frequency from the 103 to 1,305. The second phase, extending from 2016 up to the present, has been a period of rapid development, with more than 190 publications annually and an increase in citation frequency from the initial 1926 to 8,567. The number of publications and citation frequency increased rapidly (Figure 1A). Moreover, the results of the binomial regression analysis revealed that starting from 2012, the growth trend of the fitted curve was positively correlated with the year of publication (R^2^ = 0.98). The number of published articles gradually increased from 2012 to 2022, revealing a promising future growth trend in the field (Figure 1B).

### 3.2. Journals and co-cited journals

There were 103 journals that published studies on the motor development of preschoolers from 2012 to 2022. Of these journals, the top 10 in terms of the number of papers published were mostly established by research institutes and commercial companies in the United States, the United Kingdom, and Italy (Table 2). The journals with the most published articles were the *International Journal of Environmental Research and Public Health*, *Frontiers in Psychology*, and *Research in Developmental Disabilities.*

**Table 2:** High-yielding and co-cited journals (Top 10).

Rank	Journal	Number of articles published	JCR	IF	Journal	Total citation frequency	Centrality	JCR	IF
1	International Journal of Environmental Research and Public Health	83	Q2	4.614	Pediatrics	1,138	0.05	Q1	9.703
2	Frontiers in Psychology	53	Q1	4.232	Child Development	815	0.01	Q1	5.661
3	Research in Developmental Disabilities	48	Q1	3.0	Developmental Medicine and Child Neurology	799	0.03	Q2	5.366
4	Perceptual and Motor Skills	45	Q3	2.212	PLOS One	665	0	Q2	3.752
5	PLOS One	43	Q2	3.752	Research In Developmental Disabilities	619	0	Q1	3.0
6	Children-Basel	40	Q2	2.835	Child: Care, Health, and Development	568	0	Q2	2.943
7	Developmental Medicine and Child Neurology	36	Q2	5.449	Developmental Psychology	544	0	Q1	3.845
8	Early Child Development and Care	35	Q4	0.968	Journal of Science and Medicine in Sport	513	0.07	Q2	4.597
9	BMC Public Health	32	Q2	4.135	Journal of Pediatrics	507	0	Q1	6.314
10	BMC Pediatrics	31	Q3	1.909	Medicine and Science in Sports and Exercise	505	0.21	Q1	5.787

JCR, Journal Citation Reports; IF, Impact factor.

Of 747 co-cited academic journals, 10 journals were co-cited more than 500 times. All of them were established by research institutions and commercial companies in the United States and the United Kingdom (Table 2). According to the journal co-citation chart, the top three journals in terms of co-citation frequency were *Pediatrics*, *Child Development*, *Developmental Medicine, and Child Neurology*. The journals with the highest co-citation frequency and centrality in this field of study were *Medicine and Science in Sports and Exercise* (n = 505, 0.21), *Developmental Science* (n = 310, 0.27), and *Neuroscience and Biobehavioral Reviews* (n = 135, 0.23). The research reports published in these journals enables researchers to comprehend the research hotspots and frontiers in this field.

### 3.3. Analysis of disciplinary categories and coupling

The results of the disciplinary co-occurrence analysis revealed that research on international preschoolers’ motor development was not confined to the disciplines of pediatrics (n = 427), psychology (*n* = 324), and public environmental occupational health (n = 2 68). Research on this topic has also been conducted in the fields of sports sciences (*n* = 258), rehabilitation (*n* = 237), educational research (*n* = 205), clinical neurology (*n* = 140), and neurosciences (*n* = 124).

**Figure 2:**
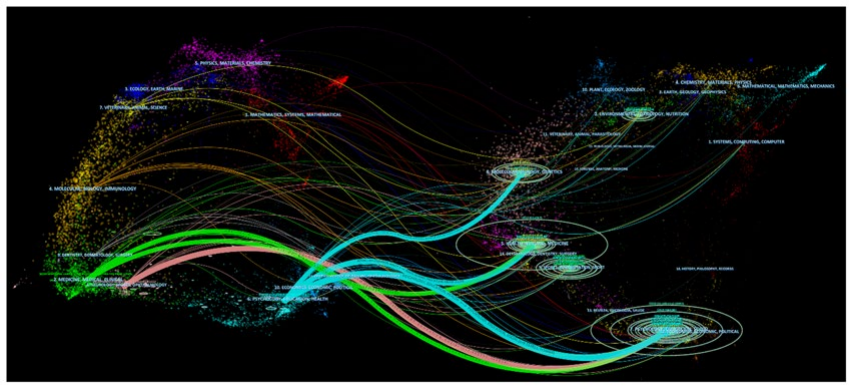
A dual-plot overlay mapping of journals showing disciplinary coupling.

With advances in science and technology, collaboration across disciplines has emerged as a new research trend. The journal biplot overlay is a new bibliometrics method that visualizes the interactions and cross-development characteristics of disciplines (16, 17). The disciplinary coupling map that is plotted divides into two major sections, connected by an arc connecting curve. The color of the arc indicates the interdisciplinary relationship path, the number indicates the frequency of interdisciplinary knowledge flow, and the thickness indicates the degree of closeness of interdisciplinary connections (17). Figure 2 illustrates the mapping of disciplinary coupling. The richer colors and areas with more arcs in the map indicate that the primary disciplinary sources in this field are more extensive and interdisciplinary. Six main citation paths (three blue, two green, and one red) are identified in the map according to the thickness of the arcs. The blue path denotes studies published in journals focusing on psychology, education, and health, which are frequently cited in studies published in journals focusing on molecular biology, genetics, medical nursing, and sociology. The green path denotes studies published in medical and clinical journals, which are frequently cited in journals focusing on health, medical nursing, psychology, education, and sociology. The red path shows published in journals focusing on neurology and kinesiology, which are frequently cited in journals focusing on psychology, education, and sociology. In conclusion, the research in the field of motor development is no longer limited to the disciplines of psychology, education, and health; it reflects a new trend of interdisciplinary collaboration with sports, molecular biology, and other fields.

## 4. Research hotspots on preschool children’s motor development

### 4.1. High-frequency keywords and clusters

**Figure 3:**
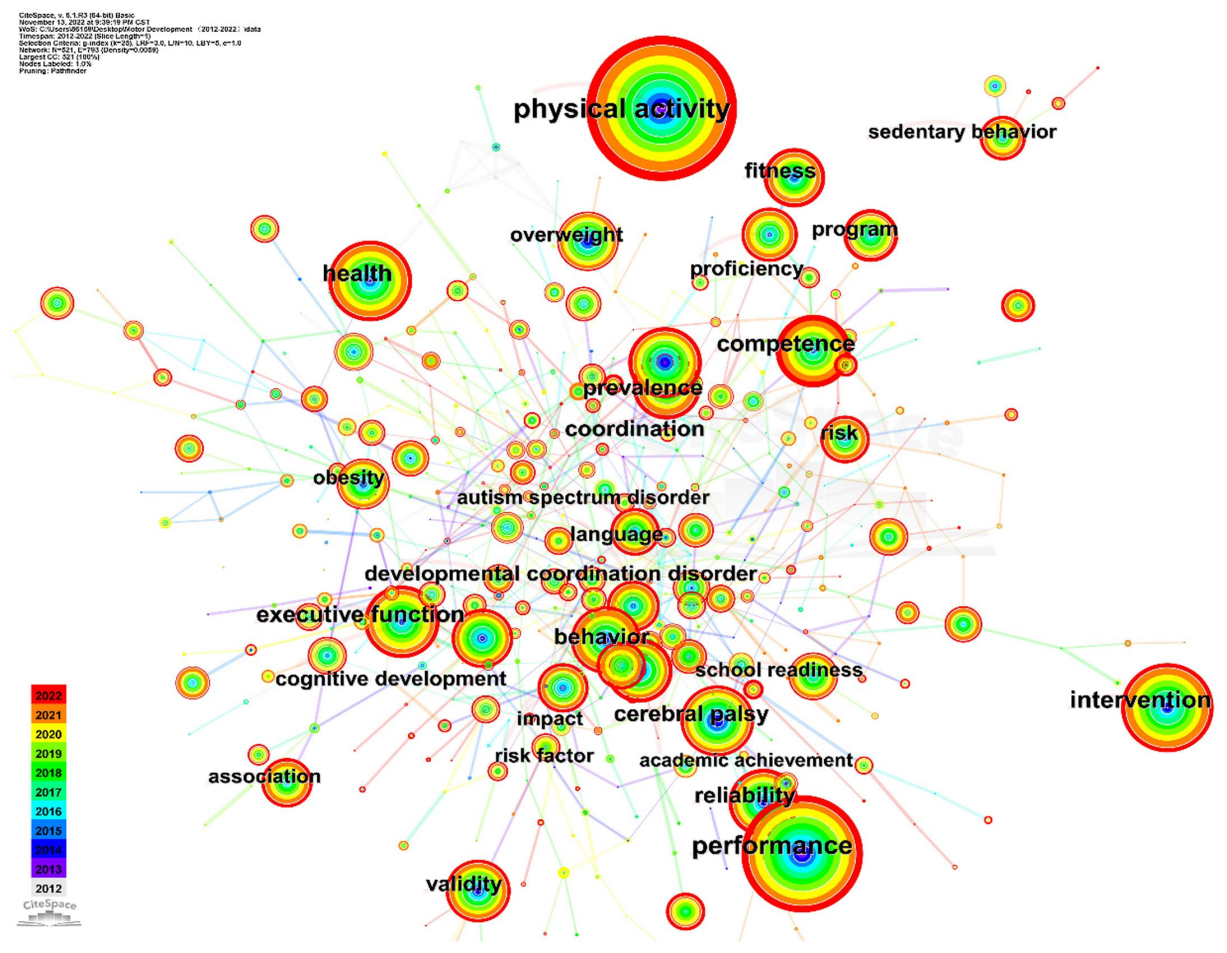
A map of co-occurring keywords.

**Figure 4:**
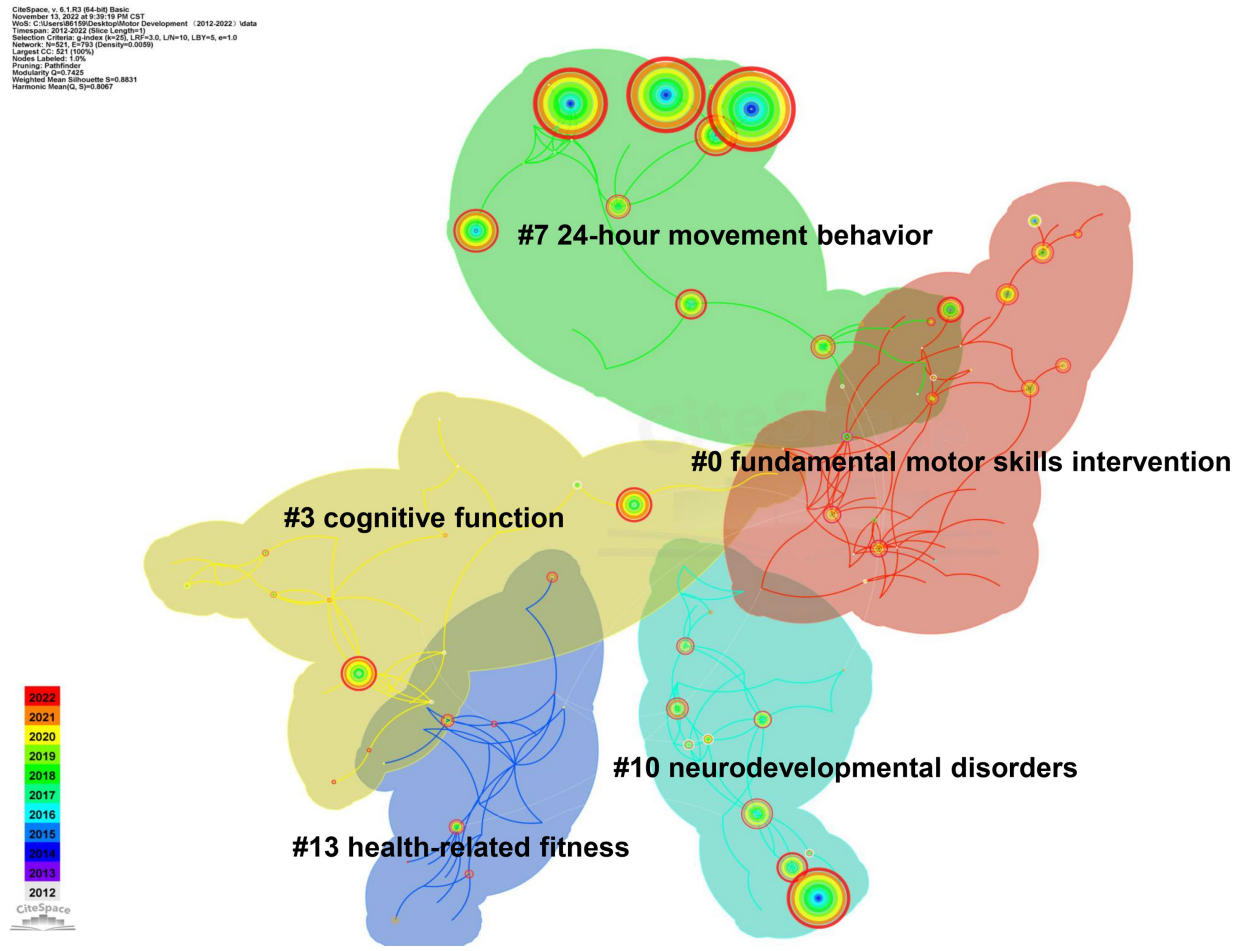
A map depicting keyword clusters.

Keywords or subject terms Are essential for summarizing The core content of academic articles, and their frequency Can indicate The trending nature of The content of a particular research field (18). CiteSpace 6.1. R4 Is a tool that analyzes research hotspots and trends In a field By conducting co-occurrence network analysis of keywords or subject terms In The cited literature (19). In this study, keywords used for 2,583 documents were analyzed To determine co-occurrence networks, and The pathfinder algorithm Was applied To Cut paths By pruning sliced networks and The merged network. Then, We combined and processed keywords with The same or overlapping meanings, such As “preschooler” and “young children” and “cognitive development” and “cognitive.” The analysis yielded a keyword co-occurrence network map containing 521 nodes and 793 links. A total of 521 keywords were extracted By removing keywords linked To retrieval strategies, such As motor development and motor skill, and 20 high-frequency and high-centricity keywords were identified (Figure 3; Table 3), which collectively represent a Hot topic In The study of motor development In preschool children. The keywords were concentrated (*Q* value = 0.74, *S* value = 0.88) within a total of 13 identified and generalized clusters, yielding five research topics. These themes included typical development of basic motor skills interventions for preschool children, research On The correlation between motor development and cognitive function and mechanisms, and The influence of 24-h motor behavior On motor development and moderating factors (Figure 4). In recent years, these research topics have been at The forefront of studies in the field of motor development.

**Table 3:** Top 20 keywords in the list by co-citation frequency and centrality.

Rank	High-frequency keywords		High-centrality keywords	
	Keywords	Frequency	Keywords	Centrality
1	Physical activity	489	Academic achievement	0.22
2	Performance	319	Low birth weight	0.16
3	Intervention	222	Association	0.14
4	Health	196	Brain	0.13
5	Executive function	165	Cerebral palsy	0.13
6	Prevalence	141	Behavior growth	0.12
7	Reliability	140	Overweight	0.12
8	Behavior	140	Typically developing children	0.12
9	Cerebral palsy	137	Autism spectrum disorder	0.11
10	Developmental coordination disorder	125	Body composition	0.11
11	Fitness	120	Developmental disability	0.11
12	Cognitive development	104	Embodied cognition	0.1
13	Overweight	100	Intelligence	0.1
14	Proficiency	98	Behavior problem	0.09
15	Program	98	Perceived competence	0.09
16	Language	95	Birth	0.08
17	Risk	91	Communication	0.08
18	achievement	90	Motivation	0.08
19	Association	90	School readiness	0.08
20	School readiness	88	Aerobic fitness	0.07

### 4.2. Research hotspots analysis via co-citation frequency/centrality with keywords

#### 4.2.1. Fundamental motor skills interventions

Our analysis of the clustering results revealed that cluster #0 mainly focused on FMS interventions. The high-frequency and high-centered keywords associated with this cluster included performance, intervention, and health, which together constituted a research hotspot.

Mastery of FMS is a prerequisite for physical activity (PA) and the development of more complex motor skills, in addition to being a critical requirement for maintaining good health during childhood and adolescence (20). Studies indicate that children begin to develop FMS at the age of 4 years, gaining proficiency by the age of 6 years. Therefore, the preschool years are a critical period for promoting FMS development (21). However, FMS development is an iterative learning process driven by a combination of physical structure or function, task constraints, and environmental changes (22) and requires instruction and reinforcement from parents, coaches, and other professionals (22). Different intervention programs can contribute to the development of FMS in preschool children, but there is wide variation in intervention outcomes. The reasons for this variation thus require further investigation (23).

In FMS intervention programs implemented for preschoolers, intervention outcomes may be influenced by variations in intervention type, models, content, and duration. Currently, FMS interventions are implemented in schools, families, or communities and are based on teacher-led, child-centered, and teacher-parent models. They may be structured or unstructured, with a single intervention lasting 30 to 50 min (24). An FMS intervention frequency of two or more interventions per week lasting 5 to 130 weeks was found to yield inconsistent results (25). One study found that interventions led by experts in the relevant field or by teachers were most effective in improving FMS levels in preschool children compared with interventions performed as free-time activities (26). While both boys and girls benefit from the interventions, boys demonstrate higher levels of positive reinforcement and stimulation in relation to object control skills, possibly because of gendered preferences that influence the selection of the intervention content (27).

FMS levels can be effectively enhanced through structured interventions, but they are more effective when the individual who implements the intervention has the necessary expertise, the possible reason is that non-expert implementers are unable to adapt the intervention content, to adapt the ongoing stimulation to the individual and context being treated. In addition, young children are difficult to maintain appropriate responses to ongoing stimulation (28). Moreover, young children’s waning interest during sessions that exceed the predetermined duration may cause a diminishing effect over time, leading to a loss of compliance and motivation (29). Interventions with a duration of 5 weeks or longer can improve FMS levels in preschoolers, but the majority of studies have only demonstrated short-term intervention effects, and data from long-term follow-up studies are lacking. Some studies have shown that the beneficial effects of FMS interventions persist for 8 to 12 weeks after the intervention (30), but their effects diminish over time and disappear completely after 12 to 48 weeks, revealing that FMS must be repeatedly taught, practiced, and reinforced (31).

In conclusion, the effects of FMS interventions vary somewhat according to the subjects’ characteristics, the intervention measures, who is performing the intervention, and modes. Therefore, further research is required to determine the optimal intervention protocol and potential mechanisms. As a next step, high-quality randomized study should be conducted to determine an optimal intervention protocol. Moreover, research on the relevant variables influencing the intervention effect (perceived motor ability, the socioeconomic environment, parental support, etc.) should be conducted. Lastly, suitable FMS intervention protocols should be designed for the target populations of children of various ages and characteristics, as well as the influencing factors.

#### 4.2.2. Underlying mechanisms mediating the association between motor skills and cognitive function

Cluster #3 focused on the relationship between motor skills and cognitive function. The focal point of this research was indicated by high-frequency and highly-centered keywords, such as cognitive development, executive function, and language, associated with this cluster. Consequently, the title of this study is “Underlying mechanisms mediating the association between motor skills and cognitive function.”

Cognitive function refers to the mental processes involved in acquiring knowledge and understanding through thought, experience, and the senses, including executive function (EF), language, intelligence, attention, perception, memory, and visuospatial ability (32). Preschool is a crucial time for rapid development of motor and cognitive skills. Changes in motor development can provide new learning opportunities to interact with their environment, targets, and caregivers, thereby promoting the development of cognitive functions. Studies have demonstrated that motor skills facilitate independence and exploration in infants and toddlers, and gross and fine motor skills during this period are predictive of subsequent language development in children (33). However, there has been limited research in this area, given the separation of motor skills and cognitive functions.

Weak to strong correlations exist between distinct types of motor skills and dimensions of cognitive function (34). Previous studies have revealed co-developmental and mutually reinforcing correlations between motor skills and EF (35). Weak correlations have been found between levels of gross motor skills and crystallized intelligence, while correlations with academic performance are unclear. There is a lack of evidence of correlations between gross muscle motor skills and short/long-term memory and working memory (33). However, weak to moderate correlations have been found between fine motor skills and fluid intelligence, short-term memory, working memory, and EF (36). However, correlations with visuospatial ability and attention, however, remain unsupported.

There has also been some debate regarding the mechanisms associated with motor skills and cognitive function in preschoolers. One theory is that multiple sclerosis and cognitive function are two distinct developmental processes that involve different brain regions (37). However, recent studies indicate that there is a strong correlation between motor skills and cognitive function, and that the mechanism may be linked to the simultaneous development of motor skills and cognitive function, with the developmental peak occurring between the ages of 5 and 10 years (38). Motor skills and cognitive functions share executive processes (sequencing, monitoring, and planning) (39). Moreover, neuroanatomical studies have demonstrated that motor skills and cognitive functions share a common extended developmental pattern and some of the same brain regions, such that the regions involved in motor skill and cognitive development (respectively the cerebellum and prefrontal cortex) are co-activated through movement and the performance of cognitive tasks. Brain structures, such as the basal ganglia, and neurotransmitters, such as dopamine, are involved in the performance of these tasks (40, 41).

Several studies have investigated the association between motor skills and cognitive function and its mechanisms, but there are still many unanswered questions remain. Future studies should attempt to determine the correlation between motor skills and cognitive function in preschool children of varying ages and characteristics. Moreover, studies should explore the factors that influence the degree of correlation, including individual characteristics (birth weight, gestation period, behavioral problems, etc.), family characteristics (education level, socioeconomic status, etc.), and school characteristics. Lastly, anatomical and biological models should be developed for elucidating the associated biological mechanisms.

#### 4.2.3. Twenty-four-hour movement behaviors and fundamental movement skills

Cluster #7 focused on the association between physical activity (PA), sedentary behavior (SB), and sleep duration (SLP) and their effects on FMS. The high-frequency and high-centered keywords associated with this cluster included PA, SB, and behavioral problems, which together constitute this research hotspot, with a primary focus on two aspects. The first is bidirectional relationships between PA and FMS and the second is combinations of PA, SB, and SLP and their associations with FMS.

##### 4.2.3.1. Bidirectional relationships between physical activity and fundamental movement skills

Lack of PA is an increasing public health issue, which impose a significant economic burden on countries worldwide. Therefore, it is essential to develop strategies to increase PA levels in preschool children, and one way of achieving this is by improving gross motor skills (42). According to the model developed by Stodden et al., there are bidirectional relationships between PA and motor competence (MC) (43). During early childhood, PA can promote MC development. However, with increasing age, the relationship between PA and MC becomes reciprocal; higher levels of PA promote MC development, while insufficient levels of MC lead to decreased PA engagement, and over time, this bidirectional relationship is reinforced (43). MC is often used as an umbrella term encompassing the various terms associated with motor development (i.e., motor proficiency, FMS, and motor coordination).

The findings of studies on the extent and direction of the association between PA and MC in preschool children are inconsistent. This association may be influenced by age, sex, various aspects of MC, and different types of PA (44). Low to moderate correlations were found between FMS, object control skills, and locomotor skills, respectively, and total physical activity in preschool children. Studies also found a weak to moderate positive correlation between FMS, object control skills, and moderate to vigorous physical activity (MVPA), which may be related to variables such as perceived motor competence (PMC) (45, 46). However, these findings are inconsistent.

The reasons for the lack of significant associations between FMS, object control and locomotor skills, and light physical activity, and the relationship between stability skills and different types of PA remain unclear. PA was not found to be associated with gross motor skills in infants aged 11 to 29 months (47). However, vigorous physical activity evidenced a weak but significant positive association with MC in children aged 5 years (42). Nevertheless, the correlation between locomotor skills and MVPA is not entirely consistent, and the relationship between stability skills and MVPA is still unclear (44). In addition, one study found that MC and PA develop separately in children aged between 2 and 6 years (48). Given insufficient evidence, the association between MC and PA in male and female children with different characteristics remains unclear.

In conclusion, the findings of previous studies do not fully support or refute a bidirectional relationship between PA and MC in early childhood, as posited by Stodden’s model. Additional RCTs or longitudinal evidence are required to investigate the temporal order, strength of association, and causality between MC and total physical activity and to establish the “dose-effect” relationship. In addition, an investigation of the mediating variables (PMC, etc.) and moderators (age, sex, maturity, etc.) that influence the association between PA and MC is necessary to provide a basis for the specification of PA protocols for children of different ages and with varying characteristics.

##### 4.2.3.2. Combinations of physical activity, sedentary behavior, and sleep duration and their associations with fundamental movement skills

As research on PA continues to evolve, it is no longer sufficient to consider individual behaviors in isolation, as individuals’ daily behaviors are confined to 24 h. Thus, PA, SB, and SLP are collectively referred to as “24-h behaviors.” PA, SB, and SLP should be considered holistically since the time spent engaged in one behavior influences other behaviors during the rest of the day, and the combination of 24-h movement behaviors may have a greater effect on the physical and mental health of children (49, 50).

Preschool children’s physical health and growth are adversely affected by insufficient PA, excessive SB, and insufficient SLP (51). Guidelines for 24-h movement behaviors recommend that a healthy preschooler’s 24-h behavior should consist of at least 3 h of PA of varying intensities, including at least 1 h of MVPA, less than 1 h of SB at a stretch, and 10 to 13 h of high-quality SLP (52). However, one study revealed that only 9.3% of preschoolers met the internationally recommended levels of 24-h movement behaviors (53). Adhering to the 24-h movement guidelines was positively associated with enhanced FMS levels among preschoolers 1 year later. Although isochronous substitution effect studies have demonstrated that MVPA isochronous substitution of light physical activity will provide positive FMS health benefits, the health benefits relating to FMS through MVPA isochronous substitution of SB are unknown and may be associated with the effect of SB on motor skills (54).

In conclusion, there is limited evidence that 24-h movement behaviors have an effect on FMS in preschoolers. Therefore, the next step is to determine the combined association between 24-h motor behaviors and different dimensions of FMS and to adjust the association between these behaviors, considered together, and after individual behaviors with FMS to determine more concisely the “dose-effect” relationship.

#### 4.2.4. Motor impairment and early intervention in children with neurodevelopmental disorders

Cluster #10 focused on motor skills in children with neurodevelopmental disorders (NDDs). Autism spectrum disorder (ASD), developmental coordination disorder, (DCD), and cerebral palsy were high-frequency and high-centered keywords associated with this cluster, constituting a research hotspot in this area. The title of this study is “Motor impairment and early interventions in children with neurodevelopmental disorders.”

The most prevalent NDDs in preschool-aged children are DCD, ASD, cerebral palsy, and attention deficit hyperactivity disorder. Epidemiological surveys indicate that approximately 15% of children aged between 3 and 17 years are affected by NDDs worldwide, with DCD, attention deficit hyperactivity disorder, and ASD being the most prevalent disorders (55). In the absence of an intervention, motor skills, language development, behavior, and learning memory continue to deteriorate in children with NDDs, typically in the form of delayed or impaired movement, impaired motor coordination, and abnormal motor posture throughout the developmental life cycle (55–57). Compared with typically developing preschoolers of the same age, children with DCD, attention deficit hyperactivity disorder, or ASD have poorer gross/fine motor skills, postural control, and motor coordination (58, 59). However, there is some heterogeneity in motor deficits among individuals with different types of NDDs. For instance, there are five distinct subtypes of children with DCD, including those whose gross motor skills are superior to their fine motor skills, those with high visuomotor integration and PMC, and those with poor kinesthetic and visual abilities (57). The characteristics and subtypes of motor skill deficits inherent to various types of NDDs in children are not yet known.

Motor impairment is prevalent in children with NDDs (60, 61), which not only has a negative impact on their daily lives but is also negatively associated with the development of cognitive skills, academic performance, and social–emotional skills (62). In addition, children with NDDs participate less frequently in PA compared to typically developing children, and this trend may persist into adulthood (63). Deficits in motor skills do not improve as children age; rather, they worsen (22). Therefore, effective intervention programs must be implemented to support the development of motor skills in children with NDDs. Intervention programs, such as physical therapy, behavioral interventions, and exercise therapy, have demonstrated positive outcomes in clinical practice.

Interventions can be therapeutic for children with NDDs of any age, but early interventions are the most effective. Currently, exercise therapy is the primary focus of early interventions for children with NDD and motor skill deficits. Exercise-based interventions can provide opportunities for children with NDDs to learn, practice, and consolidate their motor skills, resulting in higher skill levels (63). However, research on such programs is somewhat controversial. Interventions such as compulsory exercise, interventions targeting motor skills, and task-oriented training can all improve motor skills in children with NDDs (64, 65).

Although recent systematic reviews indicate that exercise-based interventions can potentially improve motor skills in children with NDDs, the effect is not statistically significant, and the quality and bias of the included literature may impact the credibility of the findings (63). Furthermore, motor interventions significantly improved the locomotor skills of preschoolers with NDDs but did not improve their object control skills (63). Two studies found that an 8-week exercise-based intervention was superior to a 4-week exercise-based intervention and that differences in the duration of single sessions (1 and 2 h) had no effect on the intervention outcome (64, 66). Nevertheless, the two studies were of low quality, and the association between single duration and effect should be interpreted with caution.

In conclusion, exercise-based interventions have the potential to improve motor impairment in children with NDDs, but the existing evidence is inconsistent. The next step is to identify the characteristics and subtypes of motor skill deficits inherent in children with various types of NDDs. Optimal intervention protocols for improving motor skills impairment in children with NDDs, as well as the variables influencing the effects of the interventions (sex, visuomotor integration ability, etc.), should be investigated to elucidate the underlying mechanisms.

#### 4.2.5. Motor competence and its effect on positive trajectories of health-related fitness in preschool children

The focus of Cluster #13 was MC and health-related fitness (HRF). Health, obesity and overweight, perceived competence, body composition, and fitness, which were high-frequency and high-centered keywords associated with this cluster, constitute the research hotspots in this area. Their combination indicated that topical issues were the relationships between MC and HRF and between weight status and MC in preschoolers.

##### 4.2.5.1. The relationship between motor competence and health-related fitness

HRF consists of cardiorespiratory endurance, muscular strength and endurance, body composition, and flexibility, is regarded as a physical and physiological factor associated with current and future health outcomes (67). One study found that children with higher MC levels during childhood were more likely to engage in physical activity, leading to improved HRF (67). Therefore, the acquisition of motor skills during childhood is one of the prerequisites for promoting HRF development in adolescence, which is crucial for maintaining good HRF in the present and future.

Only three empirical studies have investigated the relationship between MC levels and HRF in preschool children. Sigmundsson et al. (67) reported significant correlations between preschool children’s MC levels and HRF and between fine hand motor skills, locomotor skills, and HRF. However, they did not find any significant correlations between object control skills and HRF. Another study found a correlation between object control, locomotor skills, and muscular endurance in preschoolers (68). Moreover, the most recent longitudinal study found a moderate correlation between MC levels and HRF in preschoolers, with MC was a significant predictor of HRF (50).

In conclusion, MC levels in preschoolers are associated with present and future HRF. However, the majority of current studies are cross-sectional and focus on the relationship between MC and muscular strength and endurance. Nevertheless, research about other aspects of HRF (e.g., cardiorespiratory endurance, flexibility, etc.) is scant. Future research in this field should emphasize longitudinal trajectories of HRF in preschool children with different MC levels and their potential mechanisms.

##### 4.2.5.2. A causal relationship between motor competence and weight status

The preschool years are a critical period for children’s growth and development. Weight status during this period is predictive of weight status in adulthood, with behaviors associated with poor weight status persisting into adulthood (69). Therefore, preschool is a crucial time for acquiring high levels of MC and developing healthy behaviors, in addition to offering a window for obesity prevention (70).

The weight status of children is negatively correlated with their MC levels, and this association emerges during the preschool years, becoming stronger during the elementary school years (52, 71). However, the correlation between children’s weight status and the different dimensions of motor skills is unclear. Studies have found that the development of gross motor skills is slower in overweight/obese preschoolers compared with preschoolers who are not overweight. This finding is especially apparent in relation to locomotor and stability skills, but no differences have been found in fine motor skills and motor coordination (28, 72). It is currently unknown when this relationship begins to develop and the direction of its causality. In other words, it is unclear whether high body mass index (BMI) levels cause low MC levels or whether low MC levels cause high BMI.

Only two studies have investigated the temporal and causal relationship between MC and BMI. Antunes conducted a follow-up study that revealed the association between BMI and MC in preschool children increased with age (73). Another study utilized cross-lagged models to the causal relationship between weight status and multiple sclerosis in children aged 5 year, finding that a high BMI at age 5 predicted lower motor skills at ages 5 to 10, but poor motor skills at age 5 did not predict higher BMI (72). Furthermore, a recent longitudinal study found no significant correlation between BMI and preschoolers’ stability skills, speed running, or tennis toss (68), which contradicts the findings of previous studies (72, 74). This finding may be attributed to the fact that BMI does not clearly distinguish between fat mass and lean mass (75).

In conclusion, a correlation exists between MC and weight status in preschool children, but the results are inconsistent. In addition, the temporal order and causality of this correlation with various dimensions of MS remain unclear. Therefore, more high-quality RCTs and longitudinal studies should be conducted to investigate the temporal order and causality between various types of MS and obesity, utilizing different response obesity indicators (notably BMI, waist circumference, and sebum thickness).

## 5. Emerging research trends relating to preschool children’s motor development

We used CiteSpace to identify keyword bursts as a method for indicating new research trends and frontiers. The blue lines indicated the timeline and the red lines indicated the time interval of a certain research burst, that is, the burst start time and duration. The keyword burst intensity represents the frequency of keywords used in the cited literature, namely keywords that are closely followed by scholars in a certain research area over a period of time (76).

**Table 4:** Top 20 keywords with the strongest burst during the period 2012–2022.

Keywords	Year	Strength	Begin	End	2012–2022
Disorder	2012	10.89	2012	2015	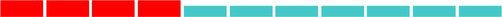
Disability	2012	4.16	2012	2018	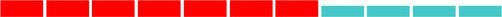
Language impairment	2012	4.12	2012	2016	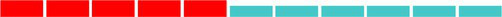
Prevention	2012	3.95	2012	2015	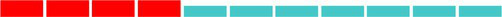
Integration	2012	3.61	2012	2013	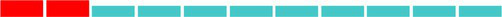
Cerebral palsy	2012	5.08	2013	2015	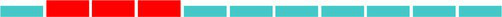
Children born	2013	3.78	2013	2016	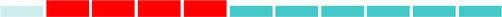
Coordination disorder	2013	3.31	2013	2017	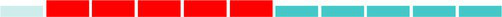
Cognition	2014	2.53	2014	2015	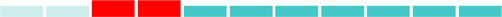
Birth weight	2015	4.53	2015	2017	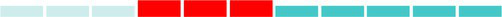
Developing country	2015	5.92	2017	2018	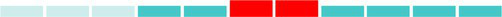
School-aged children	2017	5.86	2017	2019	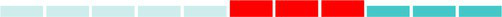
Middle-income country	2017	3.46	2017	2018	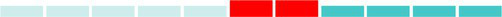
Efficacy	2018	5.41	2018	2020	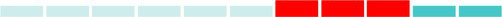
Readiness	2018	3.21	2018	2019	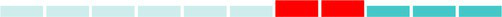
Physical development	2019	3.83	2019	2020	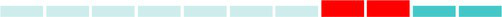
Motor proficiency	2020	3.6	2020	2022	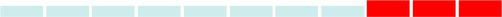
Posture	2020	3.6	2020	2022	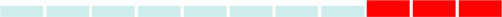
Screen time	2017	3.3	2020	2022	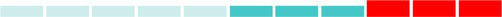
Sex	2020	2.8	2020	2022	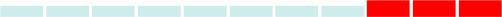

We conducted a keyword burst analysis of 2,583 studies on preschool children’s motor development included in WoSCC between 2012 and 2022 to identify the top 20 terms with the strongest burst (Table 4). We focused on keywords that could reflect the trends and frontiers of research on the motor development of preschool children. As shown in Table 4, from 2012 to 2021, the top five keywords with the strongest burst were disorder (10.89), cerebral palsy (5.08), developing country (5.92), school-aged children (5.57), and efficacy (5.41). These keywords indicate that the most important upcoming research hotspots in this field are motor impairment and early interventions in children with NDDs as well as the motor development characteristics of children with differing socioeconomic status. From 2012 to 2018, the terms disability, language impairment, prevention, cognition, birth weight, and coordination disorder were the most frequently used keywords in this field. These keywords represent the research hotspots in the field during the period 2012–2018.

Research during this period focused on the following areas: the first was the characteristics of motor skills impairment inherent in children with various types of NDDs, the best intervention program to improve motor impairment in children with NDDs, and the factors influencing the intervention effect (sex, visuomotor integration ability, etc.). The second was the correlation between motor skills and cognitive functions in preschool children of varying ages and characteristics as well as the underlying mechanisms and factors that influence the degree of correlation, such as personal characteristics (e.g., birth weight, gestation period, and behavioral problems), family characteristics (e.g., parental type, educational level, and socioeconomic status), and school characteristics. The third focus of the research was the correlation between MC and weight status in preschoolers as well as the chronology and underlying reasons for this correlation.

Research has a strong temporal component, and research hotspots are not constant. This issue can be effectively resolved using the citation burst test. The research frontiers and trends within a field can be determined by the focal research directions taken by researchers during a specific period (11, 13, 76). Between 2018 and 2022, the strongest burst keywords were developing and middle-income countries, readiness, teacher, motor proficiency, and screen time, all of which are emerging research trends relative to the previous 5 years. Future research trends in this field are likely to focus on the following topics. The first is patterns of motor development and influencing factors (e.g., age, sex, and socioeconomic status) for preschool children of varying socioeconomic status and from developing countries. The second is the chronological order, strength of association, and causal relationship between MC and SB. The third is the effect of 24-h movement behaviors on the FMS of preschoolers, the combined association of these behaviors with different dimensions of FMS, and the association with FMS after adjusting for 24-h behavior as whole and individual behavior. The fourth is developing motor proficiency and promoting the FMS of typically developing preschoolers to prepare them for school readiness.

## 6. Conclusion

Research on preschool children’s motor development has been carried out into the period of rapid development and this research will continue to advance in the future. This research field is constituted by a stable core group of authors and members of the academic community, who have made substantial contributions to its expansion. FMS interventions, the underlying mechanisms mediating the association between motor skills and cognitive functions, 24-h movement behaviors and FMS, motor impairment and early interventions in children with NDDs, and MC and the effects on positive trajectories of HRF were identified as motor development research hotspots. Moreover, the main research trends include motor development patterns in preschool children of varying socioeconomic status, MC and SB causality assessments, the effect of 24-h movement behaviors on the FMS of preschoolers, the dose-effect association with FMS after adjusting for 24-h behavior as a whole and individual behaviors, and breaking motor proficiency. In addition, we analyzed the limitations of current research was analyzed, including optimal FMS intervention programs for children of different ages and characteristics, biological mechanisms associated with motor skills and cognitive functions, and the correlation between MC and other components of HRF (e.g., cardiorespiratory endurance and flexibility). Whereas research on these topics is still preliminary, and emerge as the next set of research hot spots and trends. In summary, we reviewed and analyzed research hotspots and trends relating to motor development in preschool children, applying a bibliometric perspective. The findings of our review will contribute to broadening scholars’ research horizons and the promotion of intensive research on motor development among preschool children.

## Data availability statement

The original contributions presented in the study are included in the article/supplementary materials, further inquiries can be directed to the corresponding author.

## Author contributions

D-ML designed the study and revised the manuscript. W-JS and W-DC collected the data. SQ and XZ searched for literature and analyzed the data. J-WW and Z-CZ wrote the original draft and interpreted the results. XL provided other contribution. All authors contributed to the article and approved the submitted version.

## Funding

This work was supported by the National Social Science Fund of China (grant numbers BLA200222 and BLA220235), the Laboratory of the Sports Medicine Innovation Project of the Institute of Sports Medicine and Health, Chengdu Sport University in China (grant numbers CX21B06 and CX21C06), the Sichuan Key Laboratory of Sports Medicine 2022-2023 Open Project Grant (Project No.: YY22KX05), the Key R&D Projects of Sichuan Provincial Science and Technology Plan Project (Grant No. 2022YFS0053) and the “14th Five Year Plan” Scientific Research and Innovation Team of Chengdu Sport University (Grant No. 23CXTD02).

## Conflict of interest

The authors declare that the research was conducted in the absence of any commercial or financial relationships that could be construed as a potential conflict of interest.

## Publisher’s note

All claims expressed in this article are solely those of the authors and do not necessarily represent those of their affiliated organizations, or those of the publisher, the editors and the reviewers. Any product that may be evaluated in this article, or claim that may be made by its manufacturer, is not guaranteed or endorsed by the publisher.

## Abbreviations

FMS: fundamental motor skills
MSs: motor skills
MC: motor competence
PA: physical activity
SB: sedentary behavior
SLP: sleep duration
VPA: vigorous physical activity
MVPA: moderate to vigorous physical activity
LPA: light physical activity
EF: executive function
NDDs: neurodevelopmental disorders
ASD: autism spectrum disorder
DCD: developmental coordination disorder
PMC: perceived motor competence
HRF: health-related fitness
